# Reflection of two-dimensional surface polaritons by metallic nano-plates on atomically thin crystals 

**DOI:** 10.1515/nanoph-2022-0774

**Published:** 2023-01-30

**Authors:** Seojoo Lee, Ji-Hun Kang

**Affiliations:** School of Applied and Engineering Physics, Cornell University, Ithaca, NY 14853, USA; Department of Optical Engineering, Kongju National University, Cheonan 31080, Republic of Korea; Department of Future Convergence Engineering, Kongju National University, Cheonan 31080 , Republic of Korea

**Keywords:** graphene, nanostructures, phonon polaritons, surface polaritons, two-dimensional materials

## Abstract

Owning to their unusual optical properties, such as electrical tunability and strong spatial confinement, two-dimensional surface polaritons (2DSPs) hold great promise for deep sub-wavelength manipulation of light in a reduced low-dimensional space. Control of 2DSPs is possible by using their interaction with a boundary between two media, similar to how light behaves in three-dimensional (3D) space. The understanding of the interaction in the 2D case is still in its early stages, unlike the 3D case, as in-depth investigations are only available in a few cases including the interaction of 2DSPs with structured 2D crystals. Here, we extend the scope of our understanding to the interaction of 2DSPs with metallic nano-plates on 2D crystals, focusing on the reflection of 2DSPs. Through our rigorous model, we reveal that, for strongly confined 2DSPs having much larger momentum than free space photons, the interaction results in almost total internal reflection of 2DSPs as the radiative coupling of the 2DSPs to free space is negligible. We also find that the reflection involves an anomalous phase shift dependent on the thickness of the nano-plate, due to the temporary storing of electromagnetic energy in the evanescent waves induced near the edge of the nano-plate. Our theory predicts that the phase shift saturates to an anomalous value, 0.885*π*, as the nano-plate becomes thicker. Our work provides a detailed understanding of how to manipulate the 2DSPs by using one of the simplest nanostructures, essential for the further development of nanostructure-integrated low-dimensional devices for polariton optics.

## 1 Introduction

Macroscopic control of light is typically accomplished through interaction with a spatial boundary between two media, and intensive studies on how light interacts with the boundary have brought progressive developments in various optical devices. This idea applies not only to electromagnetic waves propagating in three-dimensional (3D) space, but also to light propagating as two-dimensional surface polaritons (2DSPs). 2DSPs, photon-coupled quasi-particles such as graphene plasmon polaritons [1–4] and phonon polaritons [5–7] residing in atomically thin 2D crystals, exhibit fascinating optical properties including strong spatial confinement and electrical tunability [1, 5, 8], [9], [10], [11], [12]. As a result, 2DSPs are thought to play an important role in deep sub-wavelength light manipulation, and detailed studies on the interactions of 2DSPs with media are required. Recent relevant investigations include the interaction of 2DSPs with the simplest structure, the edge of a 2D crystal [13, 14], involving anomalous phase shift of *π*/4 in the reflection of the 2DSPs together with unprecedented peak oscillations in the near-field interference fringes of the 2DSPs [15]. Further investigations have been made on more complicated cases such as 2DSPs reflection by corrugations [16], domain walls [17], nano-gaps in 2D crystals [18–20], and other structured surfaces [21–24].

Although the aforementioned studies provide detailed information about the interaction of 2DSPs with structured propagating space defined by a 2D crystal, understanding interaction with other types of structures, such as a metallic nanostructure on the 2D crystal [25], is still desired. The simplest case is the interaction of 2DSPs with a metal surface, which is a 2D analogue of light interaction with a metal surface in 3D space. However, the specifics of this interaction are unknown, despite the fact that it has the potential to lead to interactions with more complex nanostructures on 2D crystals. Understanding the interaction of 2DSPs with the simplest metallic structure could be a starting point for further extension to nanostructure-integrated polariton optics, just as understanding the interaction of light with structured metals in 3D space has brought plasmonic metamaterials.

In this paper, we theoretically investigate the interaction of 2DSPs with metallic nano-plates located on atomically thin crystals, focusing on the reflection of 2DSPs at a side surface of the nano-plate. We developed a rigorous model based on the diffraction of 2DSPs, and we reveal that, for strongly confined 2DSPs (i.e., 2DSP wavelengths much shorter than the free space photon wavelength), the interaction involves reflection with almost unity reflection amplitude, meaning that the reflection is very close to a total internal reflection [15]. We find that the radiative coupling of the 2DSPs to free space during the interaction is negligible, regardless of the thickness of the nano-plate. We also show that the reflection accompanies an anomalous phase shift, which is dependent on the thickness of the nano-plate. This phase shift is shown to stem from the temporary storing of electromagnetic energy in the evanescent waves induced near the edges of the nano-plate during the reflection. Our theory predicts that, as the thickness of the nano-plate increases, this phase shift saturates to a non-*π* or non-zero value, 0.885*π*, because of the evanescent waves inevitably induced in the proximity of the bottom edge of the nano-plate. Because the significance of light reflection can be found in all types of optical interferometers and resonant cavities, our understanding of the reflections of 2DSPs could lead to the development of 2DSP-based optical cavities and meta-structures integrated with 2D crystals.

## 2 Theoretical model

### 2.1 Dynamic procedure for 2DSP reflection by a metallic nano-plate

**Figure 1:**
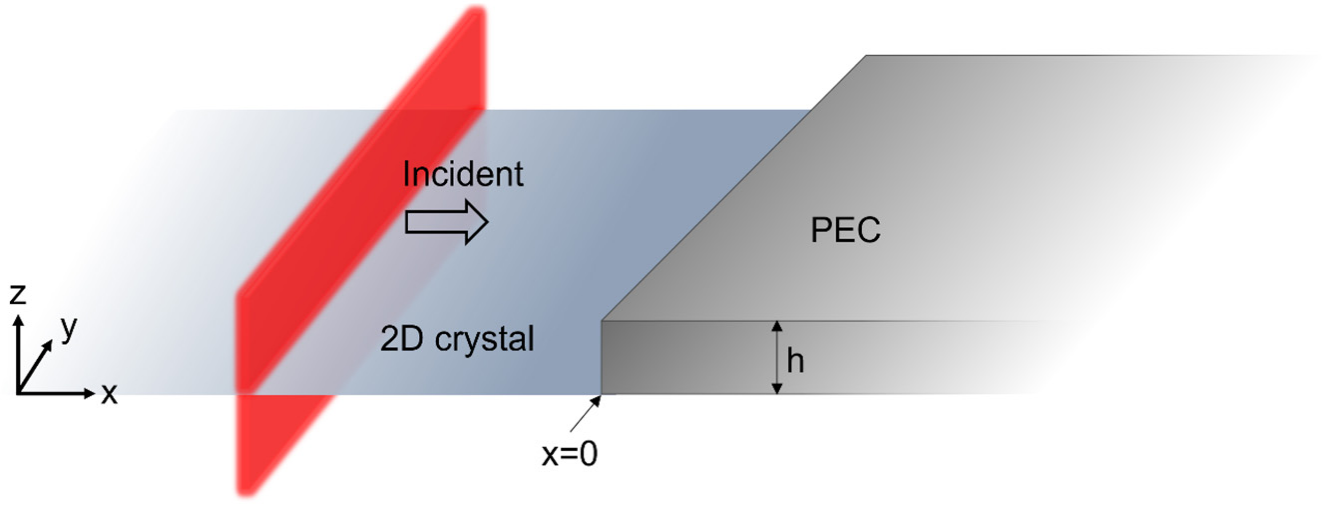
Schematic of 2D crystal and PEC nano-plate system. 2DSPs are propagating along the 2D crystal and approaching the nano-plate.

To describe the interaction of 2DSPs with a nano-plate, we consider first that there is a lossless, infinitely wide, infinitesimally thin 2D crystal of negative permittivity, located at *z* = 0 as shown in Figure 1. On the 2D crystal, a semi-infinitely wide metallic nano-plate of thickness *h* is located at *x* = 0. To simplify our problem, we assume that the nano-plate is made of a perfect electric conductor (PEC): this approximation will be justified later. The system is surrounded by a medium having positive permittivity *ε*_s_. 2DSPs, uniform in the *y*-direction, are excited by a transverse-magnetic-polarized electromagnetic wave at far-left from the nano-plate and are propagating in +*x* direction along the surface of the 2D crystal to interact with the nano-plate at *x* = 0. The interaction between the 2DSPs and the nano-plate will result in both reflection of the 2DSPs to the −*x* direction and possible radiation of the electromagnetic waves to free space, during which strong evanescent waves will be induced in the proximity of the edges of the nano-plate. This dynamic process of the interaction can be described by the expansion of the electromagnetic waves in terms of the eigenfunctions defined in corresponding spaces [15, 19]. Specifically, *y*-components of the magnetic fields can be written as (see SI for details)

(1) $$\begin{array}{l} \begin{array}{cc} {\left|H_{y}\right\rangle }_{x\leq 0} & =\left({\text{e}}^{\text{i}p_{x}x}-Re^{-ip_{x}x}\right)\left|p\right\rangle +\overset{\infty }{\underset{-\infty }{\int }}dk_{z}\alpha_{k_{z}}\left|\right.\;\left.s_{k_{z}}\right\rangle e^{-ik_{x}x}\\ & \;+\overset{\infty }{\underset{-\infty }{\int }}dk_{z}\beta_{k_{z}}\left|\right.\;\left.a_{k_{z}}\right\rangle e^{-ik_{x}x}\\ {\left|H_{y}\right\rangle }_{x\geq 0} & =\overset{\infty }{\underset{-\infty }{\int }}dk_{z}A_{k_{z}}\left|\right.\;\left.u_{k_{z}}\right\rangle {\text{e}}^{\text{i}k_{x}x}-\overset{\infty }{\underset{-\infty }{\int }}dk_{z}B_{k_{z}}\left|\right.\;\left.l_{k_{z}}\right\rangle {\text{e}}^{\text{i}k_{x}x},\\ k_{x} & \equiv \sqrt{\varepsilon_{s}k_{0}^{2}-k_{z}^{2}}. \end{array} \end{array}$$

Here, $\left|p\right\rangle$ is the eigenfunction for 2DSPs, *p*_*x*_ = 2*π*/*λ*_sp_ is the momentum of 2DSPs with *λ*_sp_ their wavelength, *ε*_s_ is the permittivity of the surrounding medium, and *R* is the reflection coefficient. $\left|\right.\;\left.s_{k_{z}}\right\rangle$ and $\left|\right.\;\left.a_{k_{z}}\right\rangle$ respectively represent symmetric and anti-symmetric eigenfunctions for unbounded modes in the 2D crystal region (*x* ≤ 0), and *α* and *β* are their amplitudes. In the nano-plate region (*x* ≥ 0), $\left|\right.\;\left.u_{k_{z}}\right\rangle$ and $\left|\right.\;\left.l_{k_{z}}\right\rangle$ represent plane wave components for the upper (*z* ≥ *h*) and lower (*z* ≤ 0) regions with corresponding amplitudes *A* and *B*, respectively. *k*_0_ is the free space photon momentum. The real-space representations of the eigenfunctions are given by

(2) $$\begin{array}{l} \begin{array}{cc} \left\langle z\left|\right.\;p\right\rangle & =\frac{z}{\left|z\right|}{\text{e}}^{\text{i}p_{z}|z|},\left\langle z\left|\right.\;s_{k_{z}}\right\rangle =\frac{1}{\sqrt{\pi }}\cos\left(k_{z}z\right),\\ \left\langle z\left|\right.\;a_{k_{z}}\right\rangle & =\frac{1}{\sqrt{\pi }}\left(\frac{z}{\left|z\right|}\frac{k_{z}}{p_{z}}\cos\left(k_{z}z\right)+i\sin\left(k_{z}z\right)\right),\\ \left\langle z\left|\right.\;u_{k_{z}}\right\rangle & =\left(e^{-ik_{z}z}+e^{-2ik_{z}h}{\text{e}}^{\text{i}k_{z}z}\right)\;for\;z\geq h,\\ \left\langle z\left|\right.\;l_{k_{z}}\right\rangle & =\left(e^{-ik_{z}z}+{\text{e}}^{\text{i}k_{z}z}\right)\;for\;z\leq 0, \end{array} \end{array}$$

with $p_{z}={\left(\varepsilon_{s}k_{0}^{2}-p_{x}^{2}\right)}^{1/2}$ . In the corresponding regions, all of those eigenfunctions are orthogonal to one another. For instance, one can examine the orthogonality between 2DSPs and the anti-symmetric radiation mode by the inner product of two eigenfunctions,

(3) $$\begin{array}{ll} \left\langle p\left|\right.\;a_{k_{z}}\right\rangle & \equiv \overset{\infty }{\underset{-\infty }{\int }}dz\left\langle p\left|\right.\;z\right\rangle \left\langle z\left|\right.\;a_{k_{z}}\right\rangle \\ & =\frac{1}{\sqrt{\pi }}\overset{\infty }{\underset{-\infty }{\int }}dz{\left(\frac{z}{\left|z\right|}{\text{e}}^{\text{i}p_{z}|z|}\right)}^{*}\\ & \;\times \left(\frac{z}{\left|z\right|}\frac{k_{z}}{p_{z}}\cos\left(k_{z}z\right)+i\sin\left(k_{z}z\right)\right)=0 \end{array}$$

where ^*^ denotes the complex conjugation.

After obtaining electric field configurations from Maxwell’s equations, one can readily apply the boundary condition at the interface at *x* = 0. Continuity of the tangential component of the magnetic field at the interface yields

(4) $$\begin{array}{ll} & \left(1-R\right)\left|p\right\rangle +\overset{\infty }{\underset{-\infty }{\int }}dk_{z}\left(\alpha_{k_{z}}\left|s_{k_{z}}\right\rangle +\beta_{k_{z}}\left|a_{k_{z}}\right\rangle \right)\\ & \;=\overset{\infty }{\underset{-\infty }{\int }}dk_{z}\left(A_{k_{z}}\left|u_{k_{z}}\right\rangle -B_{k_{z}}\left|l_{k_{z}}\right\rangle \right), \end{array}$$

and that of the electric field gives

(5) $$\begin{array}{ll} & p_{x}\left(1+R\right)\left|p\right\rangle -\overset{\infty }{\underset{-\infty }{\int }}dk_{z}k_{x}\left(\alpha_{k_{z}}\left|s_{k_{z}}\right\rangle +\beta_{k_{z}}\left|a_{k_{z}}\right\rangle \right)\\ & \;=\left\{\begin{array}{c} \overset{\infty }{\underset{-\infty }{\int }}dk_{z}k_{x}\left(A_{k_{z}}\left|u_{k_{z}}\right\rangle -B_{k_{z}}\left|l_{k_{z}}\right\rangle \right)\;\\ 0\;\;\;for\;0\leq z\leq h.\; \end{array}\right. \end{array}$$

Now, our problem on the interaction between the 2DSPs and the nano-plate is changed to fix the five undetermined coefficients, *R*, *α*, *β*, *A*, and *B*, meaning that there have to be five equations implicated in Eqs. (4) and (5). Since the coefficients are of corresponding orthogonal functions, their expression can be obtained explicitly by the dependencies between the eigenfunctions. Specifically, projections of Eq. (4) onto $\left|u_{k_{z}}\right\rangle$ and $\left|l_{k_{z}}\right\rangle$ respectively give rise to

(6) $$\begin{array}{cc} & \left(1-R\right)\left\langle u_{k_{z}}\left|\right.\;p\right\rangle +\overset{\infty }{\underset{-\infty }{\int }}dk_{\zeta }\left(\alpha_{k_{\zeta }}\left\langle u_{k_{z}}\left|\right.\;s_{k_{\zeta }}\right\rangle +\beta_{k_{\zeta }}\left\langle u_{k_{z}}\left|\right.\;a_{k_{\zeta }}\right\rangle \right)\\ & \;=4\pi A_{k_{z}},\\ & \left(1-R\right)\left\langle l_{k_{z}}\left|\right.\;p\right\rangle +\overset{\infty }{\underset{-\infty }{\int }}dk_{\zeta }\left(\alpha_{k_{\zeta }}\left\langle l_{k_{z}}\left|\right.\;s_{k_{\zeta }}\right\rangle +\beta_{k_{\zeta }}\left\langle l_{k_{z}}\left|\right.\;a_{k_{\zeta }}\right\rangle \right)\\ & \;=-4\pi B_{k_{z}}, \end{array}$$

while projections of Eq. (5) onto $\left|p\right\rangle$ , $\left|s_{k_{z}}\right\rangle$ , and $\left|a_{k_{z}}\right\rangle$ respectively bring us

(7) $$\begin{array}{cc} i\frac{p_{x}}{p_{z}}\left(1+R\right) & =\overset{\infty }{\underset{-\infty }{\int }}dk_{z}k_{z}\left(A_{k_{z}}\left\langle p\left|\right.\;u_{k_{z}}\right\rangle -B_{k_{z}}\left\langle p\left|\right.\;l_{k_{z}}\right\rangle \right),\\ -2\kappa_{x}\alpha_{k_{\zeta }} & =\overset{\infty }{\underset{-\infty }{\int }}dk_{z}k_{z}\left(A_{k_{z}}\left\langle s_{k_{\zeta }}\left|\right.\;u_{k_{z}}\right\rangle -B_{k_{z}}\left\langle s_{k_{\zeta }}\left|\right.\;l_{k_{z}}\right\rangle \right),\\ & \;-2\kappa_{x}\beta_{k_{\zeta }}\left(1-\frac{k_{\zeta }^{2}}{p_{z}^{2}}\right)\\ & =\overset{\infty }{\underset{-\infty }{\int }}dk_{z}k_{x}\left(A_{k_{z}}\left\langle a_{k_{\zeta }}\left|\right.\;u_{k_{z}}\right\rangle -B_{k_{z}}\left\langle a_{k_{\zeta }}\left|\right.\;l_{k_{z}}\right\rangle \right), \end{array}$$

with $\kappa_{x}\equiv {\left(\varepsilon_{s}k_{0}^{2}-k_{\zeta }^{2}\right)}^{1/2}$ . We now have five coupled integral equations in Eqs. (6) and (7) that are in the Fredholm form [26]. The complexity of the integral equations mainly comes from the non-orthogonal relationships between eigenfunctions in the two different regions. In order two deal with the coupled integral equations, we adopt two approximation methods, the Born approximation that gives a closed-form of a result [16, 19], and the super-lattice approximation that enables quantitatively accurate calculations [19].

### 2.2 First Born approximation

The first Born approximation simplifies the integral equations by ignoring the coupling between the radiation modes in the crystal region and the plane wave modes in the nano-plate region when their momenta are different (e.g., $\left\langle u_{k_{z}}\left|\right.s_{k_{\zeta }}\right\rangle =0$ when *k*_*z*_ ≠ *k*_*ζ*_). Then, Eq. (6) can be approximated as (see SI for details)

(8) $$\begin{array}{cc} A_{k_{z}} & \approx -\left(1-R\right)\frac{1}{4\pi }\frac{2ip_{z}}{k_{z}^{2}-p_{z}^{2}}e^{\left(ik_{z}+ip_{z}\right)h}\\ & \;+\frac{1}{4\sqrt{\pi }}e^{2ik_{z}h}\left[\alpha_{k_{z}}+\beta_{k_{z}}\left(\frac{k_{z}}{p_{z}}+1\right)\right]\\ & \;+\frac{1}{4\sqrt{\pi }}\left[\alpha_{k_{z}}+\beta_{k_{z}}\left(\frac{k_{z}}{p_{z}}-1\right)\right],\\ B_{k_{z}} & \approx -\left(1-R\right)\frac{1}{4\pi }\frac{2ip_{z}}{k_{z}^{2}-p_{z}^{2}}-\frac{1}{2\sqrt{\pi }}\alpha_{k_{z}}+\frac{1}{2\sqrt{\pi }}\beta_{k_{z}}\frac{k_{z}}{p_{z}}, \end{array}$$

and the second and the third lines of Eq. (7) are reduced to

(9) $$\begin{array}{cc} 2\alpha_{k_{z}}k_{x} & \approx -k_{x}\sqrt{\pi }\left[A_{k_{z}}\left(1+e^{-2ik_{z}h}\right)-2B_{k_{z}}\right],\\ 2\beta_{k_{z}}k_{x}\left(1-\frac{k_{z}^{2}}{p_{z}^{2}}\right) & \approx A_{k_{z}}k_{x}\sqrt{\pi }\left[\left(1+\frac{k_{z}}{p_{z}}\right)\right.\\ & \;\left.-e^{-2ik_{z}h}\left(1-\frac{k_{z}}{p_{z}}\right)\right]+2B_{k_{z}}k_{x}\sqrt{\pi }\frac{k_{z}}{p_{z}}. \end{array}$$

From the simple coupled linear equations in Eqs. (8) and (9), four coefficients *α*, *β*, *A*, and *B* can be written in terms of *R*. By substituting *A* and *B* into the first line of Eq. (7), we can obtain *R*_Born_, the reflection coefficient from the first Born approximation, in a familiar form such that

(10) $$R_{\mathrm{Born}}=\frac{W_{\mathrm{Born}}-1}{W_{\mathrm{Born}}+1},$$

where *W*_Born_ is the coupling strength given by

(11) $$\begin{array}{c} W_{\mathrm{Born}}\\ \equiv \overset{\infty }{\underset{-\infty }{\int }}dk_{z}\frac{4ip_{z}^{3}k_{x}}{\pi p_{x}{\left(k_{z}^{2}-p_{z}^{2}\right)}^{2}}\frac{\left[p_{z}\left({\text{e}}^{\text{i}k_{z}h}+e^{-ik_{z}h}\right)+k_{z}\left({\text{e}}^{\text{i}k_{z}h}-e^{-ik_{z}h}\right)\right]\left[k_{z}\left({\text{e}}^{\text{i}k_{z}h}-e^{-ik_{z}h}\right)-2p_{z}{\text{e}}^{\text{i}p_{z}h}\right]+\left(8k_{z}^{2}-6p_{z}^{2}\right)\left(e^{2ip_{z}h}+1\right)}{\left[8k_{z}p_{z}\left({\text{e}}^{\text{i}k_{z}h}+e^{-ik_{z}h}\right)+\left(8k_{z}^{2}+p_{z}^{2}\right)\left({\text{e}}^{\text{i}k_{z}h}-e^{-ik_{z}h}\right)\right]\left({\text{e}}^{\text{i}k_{z}h}-e^{-ik_{z}h}\right)+64k_{z}^{2}-32p_{z}^{2}}. \end{array}$$

**Figure 2:**
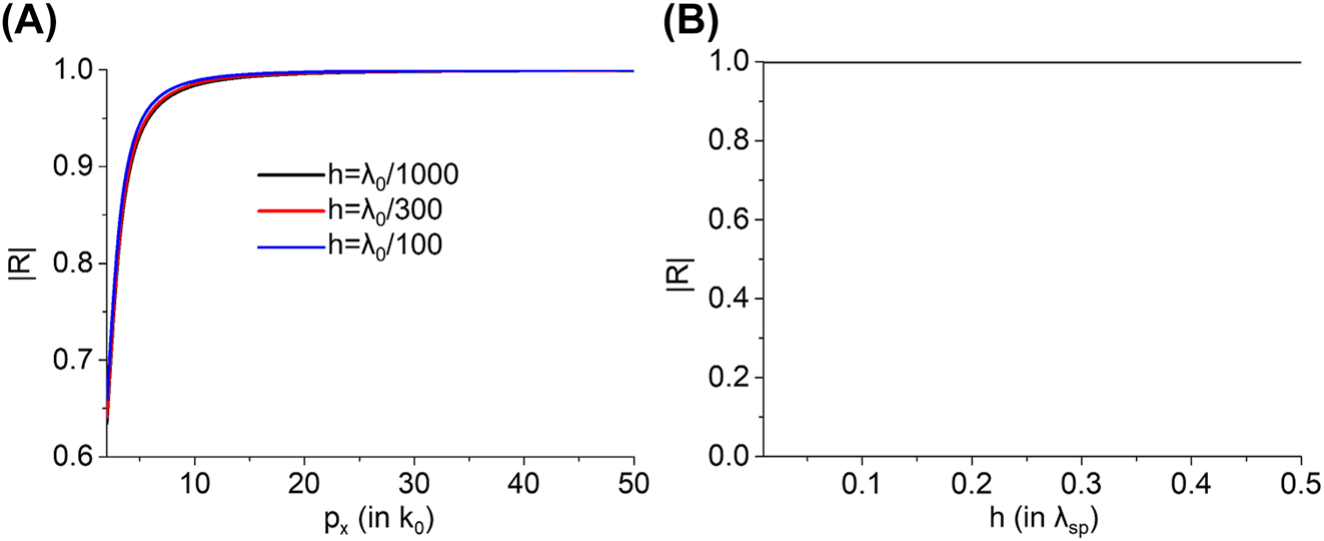
Analytically calculated amplitudes of reflected 2DSPs with (A) varying momentum of 2DSPs and (B) thickness of the nano-plate. In the calculations, the first Born approximation is used. We assumed that the system is freestanding (*ε*_s_ = 1). For (A), three exemplary thicknesses of the nano-plates, *h* = *λ*_0_/1000, *λ*_0_/300, and *λ*_0_/100, are chosen. In (B), we set *p*_*x*_ = 50*k*_0_.

Shown in Figure 2 are the amplitudes of reflected 2DSPs depending on the plasmon momentum and the thickness of the nano-plate. We can see that, regardless of the thickness of the nano-plate, the reflectance is shown to saturate to 1 as the momentum of 2DSPs *p*_*x*_ increases. This means a surprising fact that for strongly confined 2DSPs (*p*_*x*_ >> *k*_0_), the reflection is almost total internal reflection and is a lossless process, despite the fact that the nano-plate has two sharp edges from which intense diffraction of 2DSPs can emerge. The negligible radiative coupling of strongly confined 2DSPs to free space is due to a large momentum mismatch between 2DSPs and the plane wave propagating in free space: only strong evanescent waves, except for reflected 2DSPs, are induced near the edges of the nano-plate. We note that this dynamic process can be applied not only to the graphene 2DSPs of which the momentum is around 50*k*_0_ [27] but also to the fundamental phonon polariton mode in an ultrathin hexagonal boron nitride (hBN) layer as its momentum is comparable to that of the graphene 2DSPs [28].

### 2.3 Super-lattice approximation

The virtually non-radiative nature of the interaction of strongly confined 2DSPs with the nano-plate allows us to adopt the super-lattice approximation (SLA) to deal with the coupled integral equations in Eqs. (6) and (7). The basic idea of the SLA is to quantize the diffracting waves, i.e., unbounded modes and plane wave components that originally are represented as electromagnetic continua, by embedding the whole system inside a waveguide made of two parallel PEC plates, infinitely wide in the *x* direction and located at *z* = ±*d.* Here, *d* is required to be much larger than the 2DSP wavelength. Then, the PEC boundary condition $\partial_{z}\left\langle z\left|\right.\;H_{y}\right\rangle =0$ at *z* = ±*d* restricts the allowed momenta of the diffracting waves, and quantizes the electromagnetic continua through,

(12) $$\begin{array}{ll} & cos^{2}\left(\kappa_{z,n}d\right)\left(1-\frac{p_{z}^{2}}{\kappa_{z,n}^{2}}\right)=1,\\ & \eta_{z,n}=\frac{\left(n-1\right)\pi }{d},\\ & k_{z,n}=\frac{\left(n-1\right)\pi }{d-h},{\tilde{k}}_{z,n}=\frac{\left(n-1\right)\pi }{d}, \end{array}$$

where *n* is a positive integer, and *κ*_*z*,*n*_, *η*_*z*,*n*_, *k*_*z*,*n*_, and ${\tilde{k}}_{z,n}$ are quantized momenta in *z*-direction of $\left|\right.\;\left.a_{k_{z}}\right\rangle$ , $\left|\right.\;\left.s_{k_{z}}\right\rangle$ , $\left|\right.\;\left.u_{k_{z}}\right\rangle$ and $\left|\right.\;\left.l_{k_{z}}\right\rangle$ , respectively. Then, Eqs. (6) and (7) can be treated through a matrix algebra involving *N* × *N* matrices where *N* is the total number of truncated diffracting waves used in the calculation.

**Figure 3:**
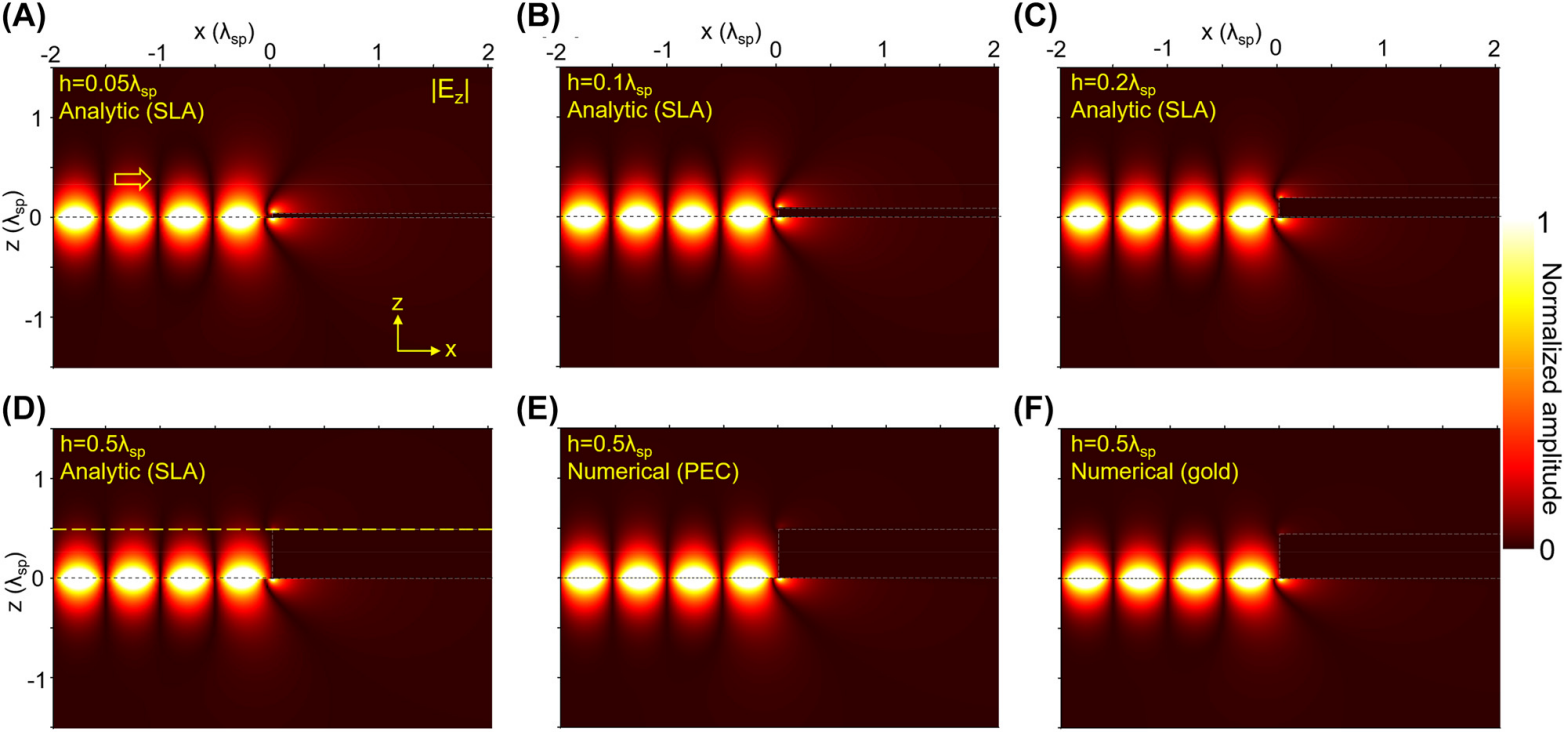
Theoretically calculated near-field maps of |*E_z_* |. (A–D) Analytically and (E, F) by 2DSP reflections bynano-plates numerically obtained maps of |*E*_*z*_| for (A) *h* = 0.05*λ*_sp_, (B) *h* = 0.1*λ*_sp_, (C) *h* = 0.2*λ*_sp_, and (D–F) *h* = 0.5*λ*_sp_. For all cases, we set *p*_*x*_ = 50*k*_0_ and *ε*_s_ = 1. For the analytic calculations, we used the SLA method, and we set *d* = 20*λ*_sp_ with *N* = 2048 the total number of the quantized diffracting waves. The numerical calculations are performed by using a commercial software COMSOL multiphysics^®^. In (E, F), we set *λ*_0_ = 6 μm, and considered 0.3 nm thick metal slab having a lossless negative permittivity *ε* = −127.3 + 0*i*. In (F), the complex permittivity of the gold is set to be *ε* = −1682.1 + 175.2*i* [29]. The horizontal yellow dashed line in (D) is where a cross-cut profile is taken to discuss in the Section 4.

Shown in Figure 3 are analytically and numerically obtained near-field maps of |*E*_*z*_| with *p*_*x*_ = 50*k*_0_ and varying thickness of the nano-plate. Owning to the almost unity 2DSP reflection amplitude ( $\left|R\right|\approx 1$ ), distinctive oscillating patterns appear on the 2D crystal with the apparent periodicity *λ*_sp_/2, resulting from the constructive and destructive interferences between the incident and reflected 2DSPs. Also, we can see that there are strong electric fields induced around the edges of the nano-plate with unique interference patterns. The induced electric fields are almost purely evanescent, so they can give rise to a specific phase shift in the reflected 2DSPs. In Figure 3A–D, the apparent strength and formation of the evanescent waves are shown to be thickness-dependent and stronger when the nano-plate is thinner, implying that the phase shift will also be dependent on the thickness and stronger in a thinner nano-plate, as we will discuss later.

To check the validity of our calculation, we performed numerical calculations and obtained field maps for *h* = 0.5*λ*_sp_ as shown in Figure 3E and F. Shown in Figure 3E is a map with a PEC nano-plate, and compared to Figure 3D, we note that there is no apparent difference between them. More importantly, Figure 3F, a map with a gold nano-plate and *λ*_0_ = 6 μm, shows an almost identical result to Figure 3E. This means that our PEC approximation is valid at least in the mid-infrared spectral regime where excitations of graphene 2DSPs and hBN phonon polaritons are usually considered.

## 3 Anomalous reflection phase shift

We have discussed the almost radiation-free reflection of strongly confined 2DSPs, inducing strong evanescent waves near the edges of the nano-plate. As addressed, this implies that the 2DSPs experience a phase shift when they get reflected at the side surface, due to the temporary storing of electromagnetic energy in the evanescent waves like the skin effect. If there are no such induced evanescent waves, the reflection phase shift would be just *π*: the meaning of the *π* phase shift is that light reflects instantaneously at the uniform interface without experiencing the skin effect.

**Figure 4:**
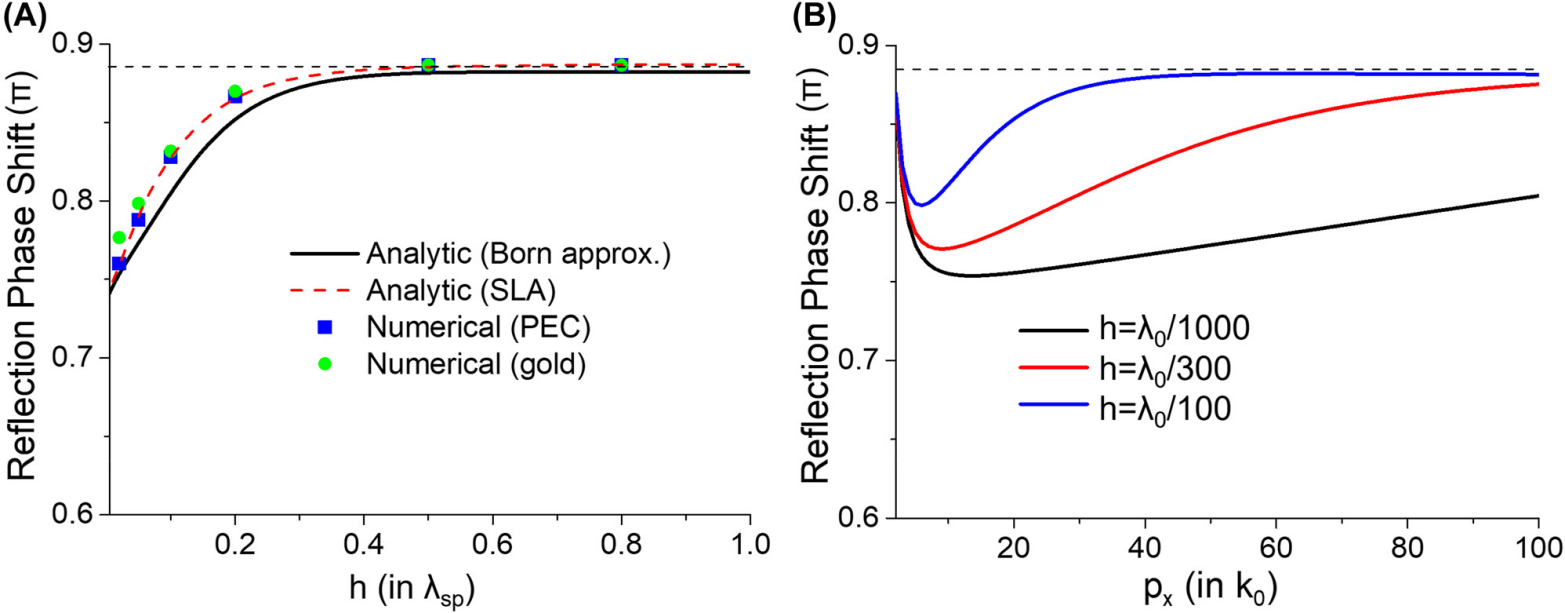
Analytically and numerically calculated phase shifts in the reflected 2DSPs. (A) Reflection phase shifts in the function of the thickness of the nano-plate with *p*_*x*_ = 50*k*_0_. For the numerical calculation, we used the same parameters as Figure 3. (B) Analytically calculated phase shifts with varying 2DSP momentum for three different thicknesses of the nano-plate. The horizontal dashed lines in (A) and (B) denote 0.885*π*. For all cases, we set *ε*_s_ = 1.

The phase shifts of 2DSPs reflection are quantitatively obtained as shown in Figure 4. In the analytic calculations, the reflection phase shift is equivalent to the argument of *R*, while in the numerical calculations the phase can be extracted by determining a position of a peak or a dip in the interference fringes of 2DSPs and measuring the distance from the side surface. In Figure 4A, we can see that the reflection phase shift is thickness-dependent, and is shown to deviate more from *π* when the nano-plate is thinner, meaning that the 2DSPs experience a stronger phase shift. This is due to that, as we can see in Figure 3, a thinner nano-plate tends to induce more evanescent waves. We note that the results from the first Born approximation are in quantitatively good agreement with the more accurate results from the SLA scheme, which are in excellent agreement with the numerical results. More importantly, we can see that the numerical results with a gold nano-plate are not much different from that with a PEC nano-plate. Only about a 5% discrepancy between the PEC and the gold results with the thinnest case (*h* = 0.02*λ*_sp_) can be observable. Considering that *h* = 0.02*λ*_sp_ with *p*_*x*_ = 50*k*_0_ corresponds to only a 6 nm thickness for 6 μm photon wavelength, the 5% difference is surprisingly small even though the complex permittivity of gold is very high (*ε* = −1682.1 + 175.2*i*) [29]. We can see that when the thickness increases beyond 6 nm, the difference between the results from the gold and PEC cases becomes insignificant.

Shown in Figure 4B are the reflection phase shifts with varying 2DSP momentum for three different thicknesses of the nano-plate. With *p*_*x*_ < 10*k*_0_, the reflection phase shifts decrease to 0.75*π*∼0.8*π* as *p*_*x*_ increases, depending on the thickness of the nano-plate. This is because, as shown in Figure 2A, as *p*_*x*_ increases, the imperfect reflection (|*R*| << 1) of weakly confined 2DSPs approaches the total internal reflection (|*R*| ≈ 1), implying that the induced evanescent waves become stronger. However, after a certain minimum, which is also dependent on *h*, the reflection phase shifts rebound and gradually approach a specific value as *p*_*x*_ increases. We observe that the minimum is the result of a competition between two factors that happens around *p*_*x*_ ≈ 10*k*_0_: the aforementioned imperfect reflection effect, which results in stronger phase shift (i.e., more deviation from *π*) with increasing *p*_*x*_, and the thickness-dependent phase shift, which becomes weaker (i.e., less deviation from *π*) with increasing *p*_*x*_. It is evident that the former predominates the latter at a lower *p*_*x*_ as the nano-plate thickens. Consequently, the minimum appears at a lower *p*_*x*_ with a thicker nano-plate and the change in the reflection phase shifts with increasing *p*_*x*_ is more pronounced with a thicker nano-plate.

As we pointed out in Figure 4A and B, the phase shifts are shown to approach a specific non-*π* value when the nano-plate gets thicker and the momentum *p*_*x*_ increases. The non-*π* phase shifts for those two cases are due to the non-vanishing evanescent waves, induced around the bottom edge of the nano-plate regardless of the thickness of the nano-plate. The specific value for the saturating phase shift can be obtained from Eqs. (10) to (11). By letting *h* → ∞, the coupling strength in Eq. (11) reduces to

(13) $$\begin{array}{l} \begin{array}{cc} \lim_{h\to \infty }W_{\text{Born}} & =-\frac{ip_{z}}{p_{x}}\frac{1}{2\pi }\overset{\infty }{\underset{-\infty }{\int }}dk_{z}\frac{2ip_{z}}{k_{z}^{2}-p_{z}^{2}}\frac{2ip_{z}}{4k_{z}^{2}-3p_{z}^{2}}k_{x}\\ & =2\left(-1+\rho \right)+\frac{4i}{\pi }\left[\tanh^{-1}\left(-\frac{ip_{z}}{p_{x}}\right)\right.\\ & \;\left.-\rho \tanh^{-1}\left(-\frac{ip_{z}}{p_{x}\rho }\right)\right], \end{array} \end{array}$$

with $\rho \equiv \sqrt{1+\varepsilon_{s}k_{0}^{2}/\left(3p_{x}^{2}\right)}$ . Then, for a very large 2DSP momentum, the coupling strength and corresponding phase shift can be approximated as

(14) $$\begin{array}{ll} & \underset{h,p_{x}\to \infty }{\lim}W_{\text{Born}}=\frac{2i}{\pi }\ln\left(\frac{4}{3}\right),\\ & \underset{h,p_{x}\to \infty }{\lim}\arg\left(R_{\text{Born}}\right)\approx 0.885\pi . \end{array}$$

This value is denoted by dashed lines in Figure 4. It is worth noting that, in Figure 4A where *p*_*x*_ = 50*k*_0_ is considered, the phase shifts at *h* = 0.5*λ*_sp_ are already very close to the saturating value 0.885*π*. For a 6 μm photon wavelength and *p*_*x*_ = 50*k*_0_, the thickness of 0.5*λ*_sp_ corresponds to 60 nm thickness which is sufficiently thin for an actual gold nano-plate. Also, according to Figure 4B, a very thin gold nano-plate of 20 nm thickness (*h* = *λ*_0_/300) will reflect graphene 2DSPs with a phase shift ranging about 0.8*π*∼0.85*π*, for a wide range of the 2DSP momentum 30*k*_0_ ≤ *p*_*x*_ ≤ 70*k*_0_ that covers most of the real-world momenta of graphene 2DSPs and hBN phonon polaritons. Therefore, in practical cases of 2DSPs operating in the mid-infrared spectral regime, it is predicted that the 2DSPs reflected by employed metallic nano-plate will experience anomalous phase shift around 0.8*π*∼0.885*π*.

## 4 Discussions

We have discussed that the non-*π* phase shift of 0.885*π* for an infinite nano-plate thickness *h* is due to the induced evanescent waves at the bottom edge. This can be verified by examining the reflection by a symmetrically located nano-plate of which the two edges are at *z* = ±*h*, suppressing strong inducement of evanescent waves near the bottom edge. Then, in our model, because the incident 2DSPs are naturally anti-symmetric in the *z*-direction, the symmetric unbounded mode $\left|s_{k_{z}}\right\rangle$ vanishes and $\left|l_{k_{z}}\right\rangle$ becomes identical to $\left|u_{k_{z}}\right\rangle$ . One can immediately show that, under this condition the first Born approximation gives the coupling strength as

$$\begin{array}{ll} W_{\text{symm}} & =\frac{p_{z}}{p_{x}}\frac{2}{\pi i}e^{2ip_{z}h}\overset{\infty }{\underset{-\infty }{\int }}dk_{z}k_{x}{\left(\frac{2ip_{z}}{k_{z}^{2}-p_{z}^{2}}\right)}^{2}\\ & \;\times {\left(6-\frac{p_{z}+k_{z}}{p_{z}-k_{z}}e^{2ik_{z}h}-\frac{p_{z}-k_{z}}{p_{z}+k_{z}}e^{-2ik_{z}h}\right)}^{-1} \end{array}$$

which goes to 0 as *h* goes to infinity, and gives *π* phase shift as expected.

It is important to take into account the effect of a surrounding medium, because in a real experimental measurement, samples are usually placed on a dielectric substrate. We point out that Eq. (14) demonstrates the independence of the 0.885*π* phase shift from the surrounding medium, meaning that the reflection phase shift is mostly determined by the momentum of 2DSPs. A substrate or a surrounding medium changes the momentum of 2DSPs, but for a given momentum, the effect of the substrate/surrounding on the phase shift is not significant as long as $|\varepsilon_{s}|k_{0}^{2}\ll p_{x}^{2}$ holds (see the Supplementary Figure in SI).

We also note that, as shown in Figure 2B, our model based on the PEC approximation exhibits an abrupt discontinuity in the thickness-dependent reflection behavior at *h* = 0. Even if the nano-plate is infinitesimally thin, the PEC approximation completely divides the nano-plate region into upper (*z* > 0) and lower (*z *< 0) regions, preventing 2DSP excitation. To accommodate the 2DSPs at *h* = 0, the two cases of *h* = 0 and *h* > 0 must be considered separately in our current model, with different basis functions. This problem could be resolved by extending our theory to deal with a metallic nano-plate with finite conductivity.

**Figure 5:**
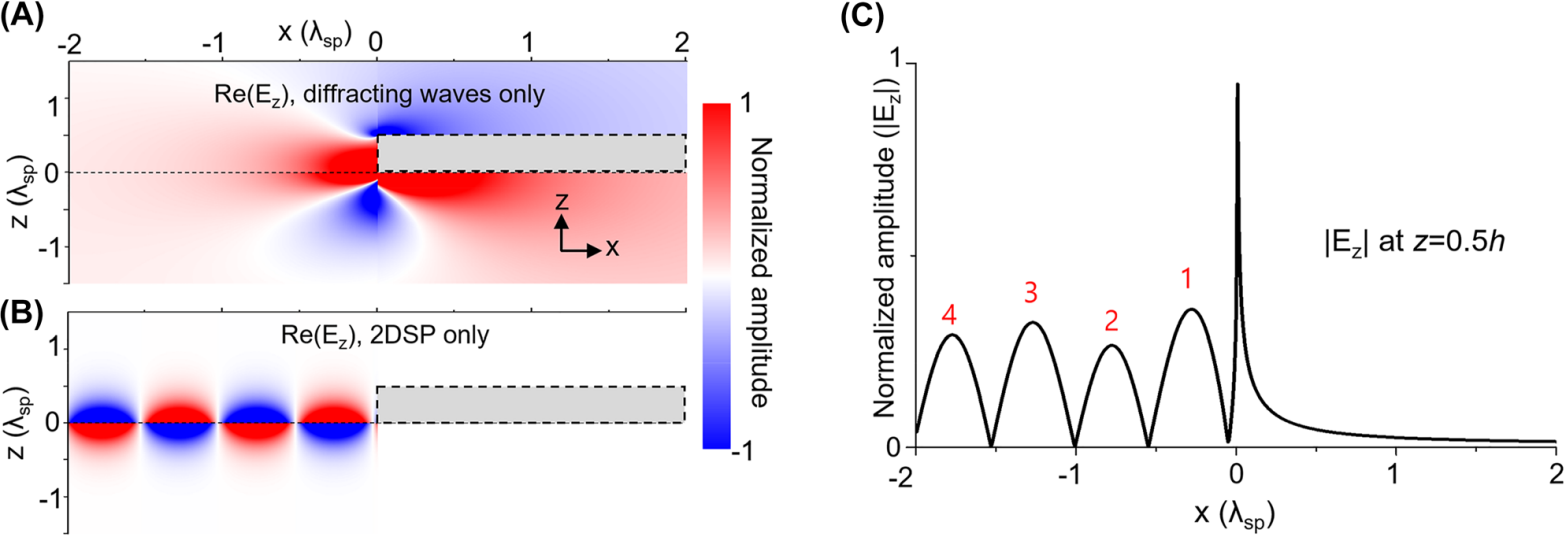
Theoretical demonstration of the even–odd peak oscillations. (A, B) Analytically calculated field maps of Re(Ez) with *h* = 0.5*λ*_sp_, and (C) crosscut profiles obtained along the horizontal yellow dashed line in Figure 3D. (A) A map of Re(Ez) for diffracting wave only, while (B) is that for the 2DSPs only. We set *ε*_s_ = 1.

Another behavior we can observe from the reflection is so-called even–odd peak oscillations, which are observed in the reflection of 2DSPs at the 2D crystal’s abrupt edge [15]. As illustrated in Figure 5, the reflection at the nano-plate also exhibits even–odd peak oscillations. The oscillations are shown to stem from the interference between the unbounded waves and 2DSPs in the crystal regions. As shown in Figure 5A, below a certain height, the unbounded waves have a constant phase along the *x*-direction, while the standing wave of 2DSP in Figure 5B has phase alternation. This yields constructive interferences for the odd-numbered peaks, and destructive ones for the even-numbered peaks, as shown in Figure 5C.

## 5 Conclusions

In this paper, we theoretically investigated the reflection of 2DSPs by the surfaces of metallic nano-plates located on atomically thin crystals. We developed a rigorous model based on the diffraction of 2DSPs, and revealed that the reflection is very close to a total internal reflection with a negligible radiative coupling of the 2DSPs to free space during the reflection regardless of the thickness of the nano-plate. We confirmed that the reflection is accompanied by an anomalous phase shift, which is dependent on the thickness of the nano-plate. The origin of this phase shift is shown to be the temporary storing of electromagnetic energy in the evanescent waves, induced near the edges of the nano-plate during the reflection. We showed that as the nano-plate becomes thicker, the phase shift saturates to 0.885*π*, not to *π* or zero, because of the evanescent waves inevitably induced near the bottom of the nano-plate. Our work provides a detailed understanding of how the 2DSPs interact with the simplest metallic nanostructures, which can be extended progressively to further development of nanostructure-integrated low-dimensional devices for polariton optics.

## Supplementary Material

Supplementary Material Details
