# Supplementary material for: Reflection of two-dimensional surface polaritons by metallic nano-plates on atomically thin crystals
Source: Nanophotonics. 2023 Jan 30;12(13):2573–81. doi: 10.1515/nanoph-2022-0774 (PMC11501456; doi:10.1515/nanoph-2022-0774)
Supplement: Supplementary file 1 — Supplementary Material Details [file j_nanoph-2022-0774_suppl_001.pdf]

# Supporting Information for
# "Reflection of two-dimensional surface polaritons by metallic nano-plates on atomically thin crystals"

Seojoo Lee[1] and Ji-Hun Kang[2,3,4,*]

[1]School of Applied Engineering Physics, Cornell University, Ithaca, NY 14853, USA

[2]Department of Optical Engineering, Kongju National University, Cheonan 31080, Korea

[3]Department of Future Convergence Engineering, Kongju National University, Cheonan 31080, Korea

[4]Institute of Application and Fusion for Light, Kongju National University, Cheonan 31080, Korea

* Corresponding author: jihunkang@kongju.ac.kr

# Analytic theory on the interaction of two-dimensional (2D) surface polaritons (SPs) with metallic nano-plate

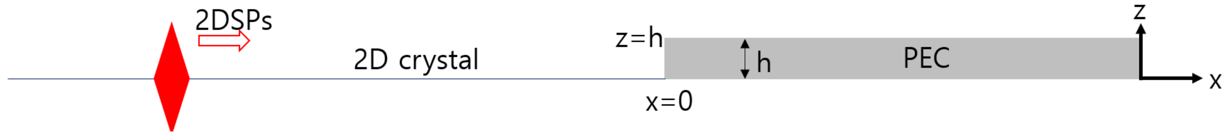


**Fig. S1**. Schematic of 2D crystal and PEC nano-plate system.

## 1. Eigenfunctions in the 2D crystal region

In order to describe the interaction of two-dimensional surface polaritons (2DSPs) with metallic nano-block, we assume that there is an infinitely wide, infinitesimally thin 2D crystal of negative permittivity $\varepsilon_m$ as shown in Fig. S1. A semi-finitely wide nano-plate of thickness $h$ is located at $x$=0. The whole system is surrounded by a medium possessing positive dielectric constant $\varepsilon_s$ greater than 1. To simplify the problem, we assume that the nano-plate is made of a perfect electric conductor (PEC), and that the permittivity of the 2D crystal is pure-real so that the system is lossless. We consider the 2DSPs are excited by transverse-magnetic field ($E_x$, $E_z$, and $H_y$). It has been shown that the 2DSPs, propagating along the $x$-direction as a surface-bound wave on the 2D crystal, can be described by the $z$-component of the electric field, of which the real-space representation of the eigenfunction is given by [1, 2]

$$\langle z | p \rangle = \frac{z}{|z|} e^{ip_z|z|} \qquad \text{(s1)}.$$

Here, $p_z = \sqrt{\varepsilon_s k_0^2 - p_x^2}$ with $p_x$ the momentum of the 2DSPs in the $x$-direction and $k_0$ the free space photon momentum. Note that the eigenfunction is anti-symmetric in the $z$-direction. In the 2D crystal region, there are two more eigenfunctions that are not surface-bound but are orthogonal to $|p\rangle$. These are called radiation modes [3] or unbounded modes [2]. They can be obtained by using the plane wave expansion method and applying the electromagnetic boundary conditions at $z$=0. The real-space representations of these unbounded modes are

$$\begin{aligned}\langle z|s_{k_z}\rangle &= \frac{1}{\sqrt{\pi}}\cos(k_z z) \quad (\text{symmetric mode}) \\ \langle z|a_{k_z}\rangle &= \frac{1}{\sqrt{\pi}}\left(\frac{z}{|z|}\frac{k_z}{p_z}\cos(k_z z)+i\sin(k_z z)\right) \quad (\text{anti-symmetric mode})\end{aligned} \qquad (\text{s2}).$$

Here, $k_z$ is the momentum of the unbounded modes in the $z$-direction. Unlike the surface-bound mode $|p\rangle$ which is uniquely defined, $|s_{k_z}\rangle$ and $|a_{k_z}\rangle$ can have continuous momentum $k_z$, meaning that the unbounded modes generally form electromagnetic continua. Together with $|p\rangle$, $|s_{k_z}\rangle$ and $|a_{k_z}\rangle$ form a complete orthogonal set of eigenfunctions through the orthogonal conditions such that

$$\begin{aligned}&\langle p|a_{k_z}\rangle \equiv \int_{-\infty}^{\infty} dz \langle p|z\rangle\langle z|a_{k_z}\rangle = \frac{1}{\sqrt{\pi}}\int_{-\infty}^{\infty} dz\left(\frac{z}{|z|}e^{ip_z|z|}\right)^{*}\left(\frac{z}{|z|}\frac{k_z}{p_z}\cos(k_z z)+i\sin(k_z z)\right)=0, \\ &\langle p|s_{k_z}\rangle = \langle a_{k_z}|s_{k_z}\rangle = 0, \\ &\langle s_{k_\zeta}|s_{k_z}\rangle = \delta(k_z+k_\zeta)+\delta(k_z-k_\zeta), \\ &\langle a_{k_\zeta}|a_{k_z}\rangle = \left(1-\frac{k_z^2}{p_z^2}\right)\left[\delta(k_z-k_\zeta)-\delta(k_z+k_\zeta)\right], \quad \langle p|p\rangle = \frac{i}{p_z}\end{aligned} \qquad (\text{s3}).$$

Every possible electromagnetic field can be described in terms of a unique linear combination of those orthogonal eigenfunctions. Therefore, electromagnetic field distribution resulting from the interaction between 2DSPs and the edge of the nano-plate can be written as

$$\begin{aligned}|H_y\rangle_{x\le 0} &= \left(e^{ip_x x}-Re^{-ip_x x}\right)|p\rangle + \int_{-\infty}^{\infty} dk_z \alpha_{k_z}|s_{k_z}\rangle e^{-ik_x x} + \int_{-\infty}^{\infty} dk_z \beta_{k_z}|a_{k_z}\rangle e^{-ik_x x}, \\ |E_z\rangle_{x\le 0} &= \frac{1}{\omega\varepsilon_s\varepsilon_0}\left[-p_x\left(e^{ip_x x}+Re^{-ip_x x}\right)|p\rangle + \int_{-\infty}^{\infty} dk_z \alpha_{k_z}k_x|s_{k_z}\rangle e^{-ik_x x} + \int_{-\infty}^{\infty} dk_z \beta_{k_z}k_x|a_{k_z}\rangle e^{-ik_x x}\right]\end{aligned} \qquad (\text{s4}),$$

with $k_x = \sqrt{\varepsilon_s k_0^2 - k_z^2}$, $R$ the reflection coefficient of the 2DSPs, $\alpha_{k_z}$ and $\beta_{k_z}$ the amplitudes of symmetric and anti-symmetric unbounded modes, respectively.

**2. Eigenfunctions in the nano-plate region**

In the nano-plate region, the corresponding eigenfunctions are simply the combination of plane waves propagating in the $\pm z$ directions and satisfying PEC boundary conditions at $z$=0 and $z$=$h$. One can obtain the eigenfunctions in the plane wave forms such that

$$\langle z | u_{k_z} \rangle = \left( e^{-ik_z z} + e^{-2ik_z h} e^{ik_z z} \right) \text{ for } z \geq h,$$
$$\langle z | l_{k_z} \rangle = \left( e^{-ik_z z} + e^{ik_z z} \right) \text{ for } z \leq 0 \qquad \text{(s5).}$$

Like the unbounded modes in the 2D crystal region, the eigenfunctions in the nano-plate region also can have continuous momentum $k_Z$, and form electromagnetic continua in general. The electromagnetic field configurations in the nano-plate region can be written as

$$|H_y\rangle_{x\geq 0} = \int_{-\infty}^{\infty} dk_z A_{k_z} |u_{k_z}\rangle e^{ik_x x} - \int_{-\infty}^{\infty} dk_z B_{k_z} |l_{k_z}\rangle e^{ik_x x},$$
$$|E_z\rangle_{x\geq 0} = -\left[ \frac{1}{\omega \varepsilon_s \varepsilon_0} \int_{-\infty}^{\infty} dk_z A_{k_z} k_x |u_{k_z}\rangle e^{ik_x x} - \int_{-\infty}^{\infty} dk_z B_{k_z} k_x |l_{k_z}\rangle e^{ik_x x} \right] \qquad \text{(s6),}$$

with $A_{k_z}$ and $B_{k_z}$ the amplitudes of plane wave components in the upper and lower regions of the nano-plate, respectively. The inner products of the two eigenfunctions give

$$\langle u_{k_z} | l_{k_z} \rangle = 0,$$
$$\langle u_{k_\zeta} | u_{k_z} \rangle = 2\pi\delta(k_z - k_\zeta) + 2\pi e^{2ik_\zeta h} \delta(k_z + k_\zeta), \qquad \text{(s7).}$$
$$\langle l_{k_\zeta} | l_{k_z} \rangle = 2\pi\delta(k_z - k_\zeta) + 2\pi\delta(k_z + k_\zeta)$$

### 3. Dependencies between eigenfunctions in the two regions

The electromagnetic coupling at an interface is determined by the dependencies between eigenfunctions defined in two different regions. In our case, the incident 2DSPs can be coupled to the plane wave components in the PEC regions owing to the non-vanishing dependencies between $|p\rangle$ and $|u_{k_z}\rangle$ and $|l_{k_z}\rangle$, resulting in the recoupling of $|u_{k_z}\rangle$ and $|l_{k_z}\rangle$ to the unbounded modes $|s_{k_z}\rangle$ and $|a_{k_z}\rangle$ in the 2D crystal region due to the same reason. Specific functional dependencies are summarized in the followings:

$$\left\langle u_{k_z} \middle| p \right\rangle = -\frac{2ip_z}{k_z^2 - p_z^2} e^{(ik_z + ip_z)h}, \quad \left\langle l_{k_z} \middle| p \right\rangle = \frac{2ip_z}{k_z^2 - p_z^2},$$
$$\left\langle l_{k_\zeta} \middle| s_{k_z} \right\rangle = \sqrt{\pi}\left[\delta\left(k_z - k_\zeta\right) + \delta\left(k_z + k_\zeta\right)\right],$$
$$\left\langle l_{k_\zeta} \middle| a_{k_z} \right\rangle = -\frac{1}{\sqrt{\pi}} \frac{2ik_z}{k_z^2 - k_\zeta^2} - \sqrt{\pi}\frac{k_z}{p_z}\left[\delta\left(k_z - k_\zeta\right) + \delta\left(k_z + k_\zeta\right)\right],$$
$$\left\langle u_{k_\zeta} \middle| s_{k_z} \right\rangle$$
$$= \frac{1}{2\sqrt{\pi}}\left[\frac{2ik_z}{k_z^2 - k_\zeta^2}\left(e^{ih\left(k_z + k_\zeta\right)} - e^{-ih\left(k_z - k_\zeta\right)}\right) + \pi\left(1 + e^{2ik_\zeta h}\right)\left(\delta\left(k_z - k_\zeta\right) + \delta\left(k_z + k_\zeta\right)\right)\right], \qquad \text{(s8)}.$$
$$\left\langle u_{k_\zeta} \middle| a_{k_z} \right\rangle$$
$$= \frac{1}{2\sqrt{\pi}} \frac{k_z}{p_z}\left[\frac{2ik_z}{k_z^2 - k_\zeta^2}\left(e^{ih\left(k_z + k_\zeta\right)} - e^{-ih\left(k_z - k_\zeta\right)}\right) + \pi\left(1 + e^{2ik_\zeta h}\right)\left(\delta\left(k_z - k_\zeta\right) + \delta\left(k_z + k_\zeta\right)\right)\right]$$
$$+ \frac{1}{2\sqrt{\pi}}\left[\frac{2ik_z}{k_z^2 - k_\zeta^2}\left(e^{ih\left(k_z + k_\zeta\right)} + e^{-ih\left(k_z - k_\zeta\right)}\right) + \pi\left(1 - e^{2ik_\zeta h}\right)\left(\delta\left(k_z + k_\zeta\right) - \delta\left(k_z - k_\zeta\right)\right)\right]$$

**4. Boundary conditions and the coupled integral equations**

Now we have all the ingredients to see how the 2DSPs interact with the nano-plate. This can be done by applying the boundary conditions of the tangential components of electromagnetic waves at the edge of the nano-plate ($x$=0). Specifically, Eq. (s4) and (s6) must satisfy the continuity requirements such that

$$\left(1 - R\right)\left| p \right\rangle + \int_{-\infty}^{\infty} dk_z \left(\alpha_{k_z}\left| s_{k_z} \right\rangle + \beta_{k_z}\left| a_{k_z} \right\rangle\right) = \int_{-\infty}^{\infty} dk_z \left(A_{k_z}\left| u_{k_z} \right\rangle - B_{k_z}\left| l_{k_z} \right\rangle\right),$$
$$p_x\left(1 + R\right)\left| p \right\rangle - \int_{-\infty}^{\infty} dk_z k_x \left(\alpha_{k_z}\left| s_{k_z} \right\rangle + \beta_{k_z}\left| a_{k_z} \right\rangle\right) = \begin{cases} \int_{-\infty}^{\infty} dk_z k_x \left(A_{k_z}\left| u_{k_z} \right\rangle - B_{k_z}\left| l_{k_z} \right\rangle\right) \\ 0 \quad \text{for } 0 \le z \le h \end{cases} \qquad \text{(s9)}.$$

Projections of the first line of Eq. (s9) onto $\left| u_{k_z} \right\rangle$ and $\left| l_{k_z} \right\rangle$ respectively give rise to

$$\left(1 - R\right)\left\langle u_{k_z} \middle| p \right\rangle + \int_{-\infty}^{\infty} dk_\zeta \left(\alpha_{k_\zeta}\left\langle u_{k_z} \middle| s_{k_\zeta} \right\rangle + \beta_{k_\zeta}\left\langle u_{k_z} \middle| a_{k_\zeta} \right\rangle\right) = 4\pi A_{k_z},$$
$$\left(1 - R\right)\left\langle l_{k_z} \middle| p \right\rangle + \int_{-\infty}^{\infty} dk_\zeta \left(\alpha_{k_\zeta}\left\langle l_{k_z} \middle| s_{k_\zeta} \right\rangle + \beta_{k_\zeta}\left\langle l_{k_z} \middle| a_{k_\zeta} \right\rangle\right) = -4\pi B_{k_z} \qquad \text{(s10)},$$

while projections of the second line of Eq. (s9) onto $\left| p \right\rangle$, $\left| s_{k_z} \right\rangle$ and $\left| a_{k_z} \right\rangle$ respectively bring us

$$i\frac{p_x}{p_z}(1+R)=\int_{-\infty}^{\infty}dk_z k_z\left(A_{k_z}\langle p|u_{k_z}\rangle-B_{k_z}\langle p|l_{k_z}\rangle\right),$$

$$-2\kappa_x\alpha_{k_\zeta}=\int_{-\infty}^{\infty}dk_z k_z\left(A_{k_z}\langle s_{k_\zeta}|u_{k_z}\rangle-B_{k_z}\langle s_{k_\zeta}|l_{k_z}\rangle\right), \qquad \text{(s11)},$$

$$-2\kappa_x\beta_{k_\zeta}\left(1-\frac{k_\zeta^2}{p_z^2}\right)=\int_{-\infty}^{\infty}dk_z k_x\left(A_{k_z}\langle a_{k_\zeta}|u_{k_z}\rangle-B_{k_z}\langle a_{k_\zeta}|l_{k_z}\rangle\right)$$

where $\kappa_x=\sqrt{\varepsilon_s k_0^2-k_\zeta^2}$. Given in Eq. (s10) and (s11) are coupled integral equations that are in the Fredholm form.

## 5. First Born approximation

One method to solve the coupled integral equations in Eq. (s10) and (s11) is to employ the first Born approximation, suppressing the coupling between the unbounded modes and the plane wave modes when the momenta of the eigenfunctions are different. Specifically, the three dependencies between the eigenfunctions in Eq. (s8) can be approximated as

$$\langle l_{k_\zeta}|a_{k_z}\rangle\approx-\sqrt{\pi}\frac{k_z}{p_z}\left[\delta(k_z-k_\zeta)+\delta(k_z+k_\zeta)\right],$$

$$\langle u_{k_\zeta}|s_{k_z}\rangle\approx\frac{\sqrt{\pi}}{2}\left[\left(1+e^{2ik_\zeta h}\right)\left(\delta(k_z-k_\zeta)+\delta(k_z+k_\zeta)\right)\right], \qquad \text{(s12)}.$$

$$\langle u_{k_\zeta}|a_{k_z}\rangle\approx\frac{\sqrt{\pi}}{2}\left[\left(\left(\frac{k_z}{p_z}-1\right)+\left(\frac{k_z}{p_z}+1\right)e^{2ik_\zeta h}\right)\delta(k_z-k_\zeta)+\left(\left(\frac{k_z}{p_z}+1\right)+\left(\frac{k_z}{p_z}-1\right)e^{2ik_\zeta h}\right)\delta(k_z+k_\zeta)\right]$$

Then, by putting Eq. (s12) into Eq. (s10) and (s11), one can find the approximated coupled integral equations shown in Eq. (8) and (9) in the main text.

**6. Supplementary figure**

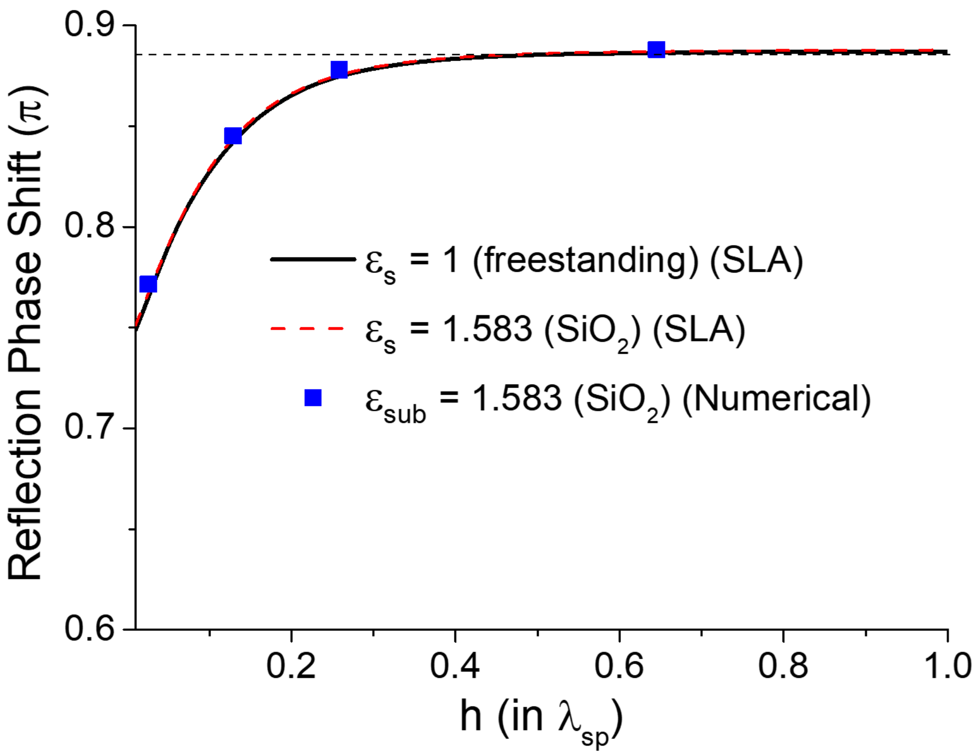


**Fig. S2**. Analytically and numerically obtained reflection phase shifts with $SiO_2$ surrounding (analytic) or $SiO_2$ substrate (numerical). $\varepsilon_{sub}$ denotes the permittivity of the substrate. We set $p_x$=50$k_0$.

**References**


[1] Alù, Andrea, Engheta, N. *J. Opt. Soc. Am. B* **23**, 571 (2006)

[2] J. H. Kang, S. Wang *et al.*, Nano Lett. **17**, 1768 (2017).

[3] D. Marcuse, *Theory of Dielectric Optical Waveguides* (Academic, 1974).
